# Supplementary figures and images for: Efficient modelling of infectious diseases in wildlife: A case study of bovine tuberculosis in wild badgers
Source: PLoS Comput Biol. 2024 Nov 19;20(11):e1012592. doi: 10.1371/journal.pcbi.1012592 (PMC11614247; doi:10.1371/journal.pcbi.1012592)

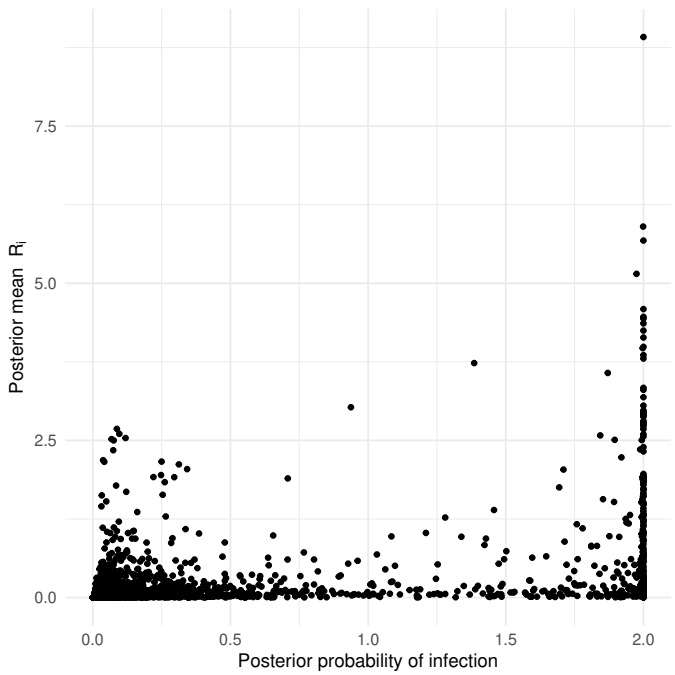

Supplement: S1 Fig — (PDF) [file pcbi.1012592.s002.pdf]

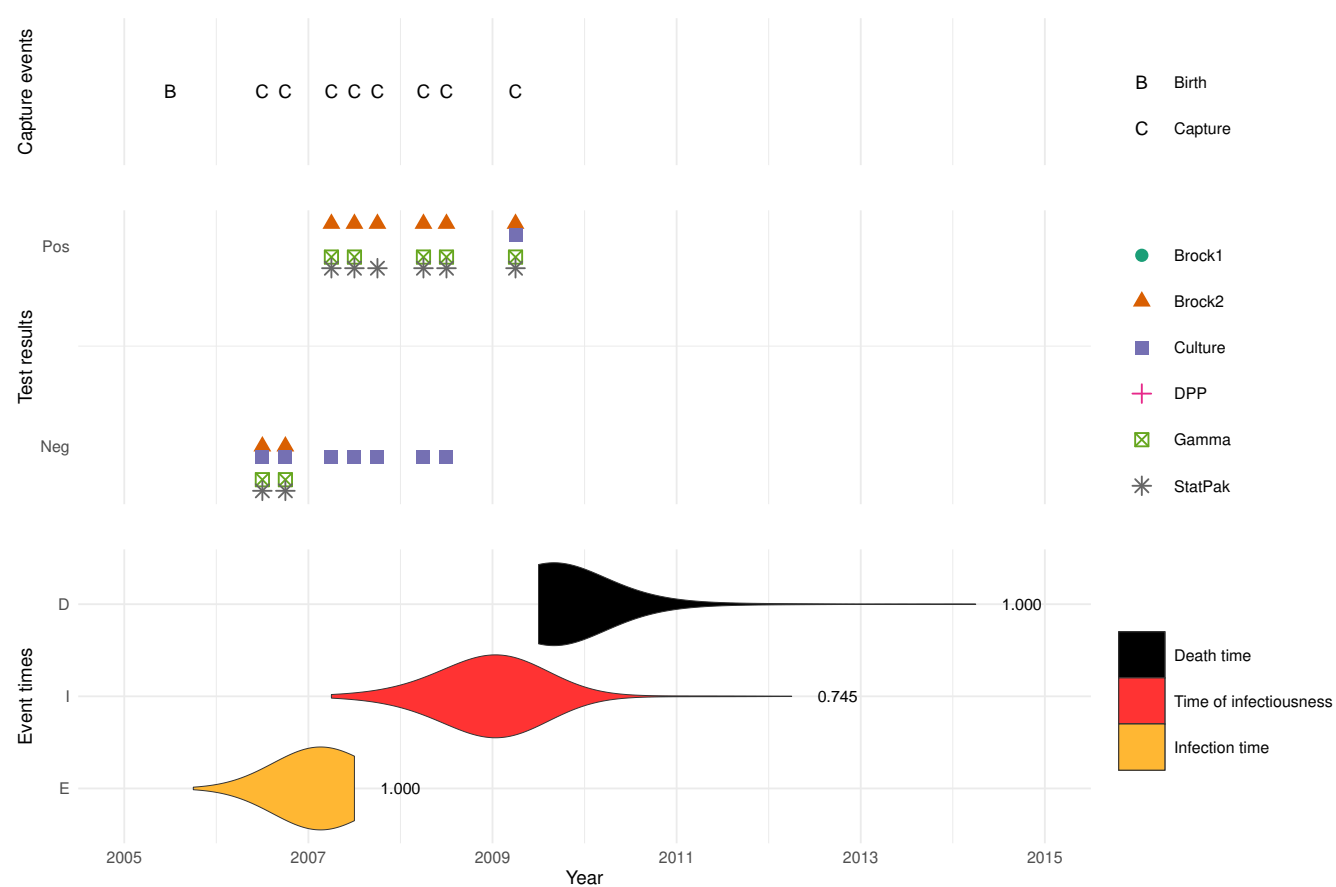

Supplement: S2 Fig — Densities are the conditional posteriors for the event time given that the event occurred. (PDF) [file pcbi.1012592.s003.pdf]

Posterior distribution of the number of individuals in each infection state

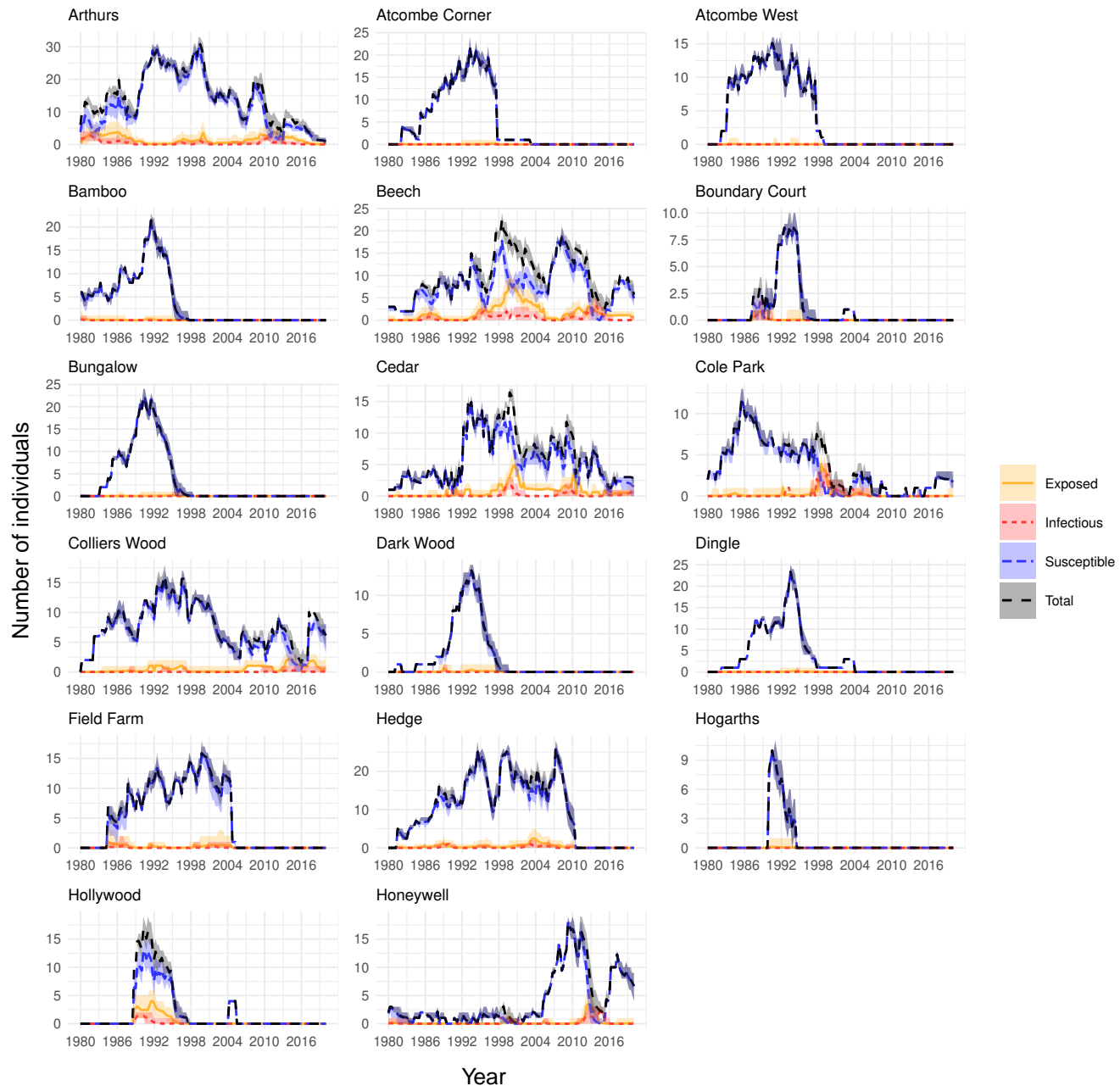

Supplement: S3 Fig — (PDF) [file pcbi.1012592.s004.pdf]

Posterior distribution of the number of individuals in each infection state

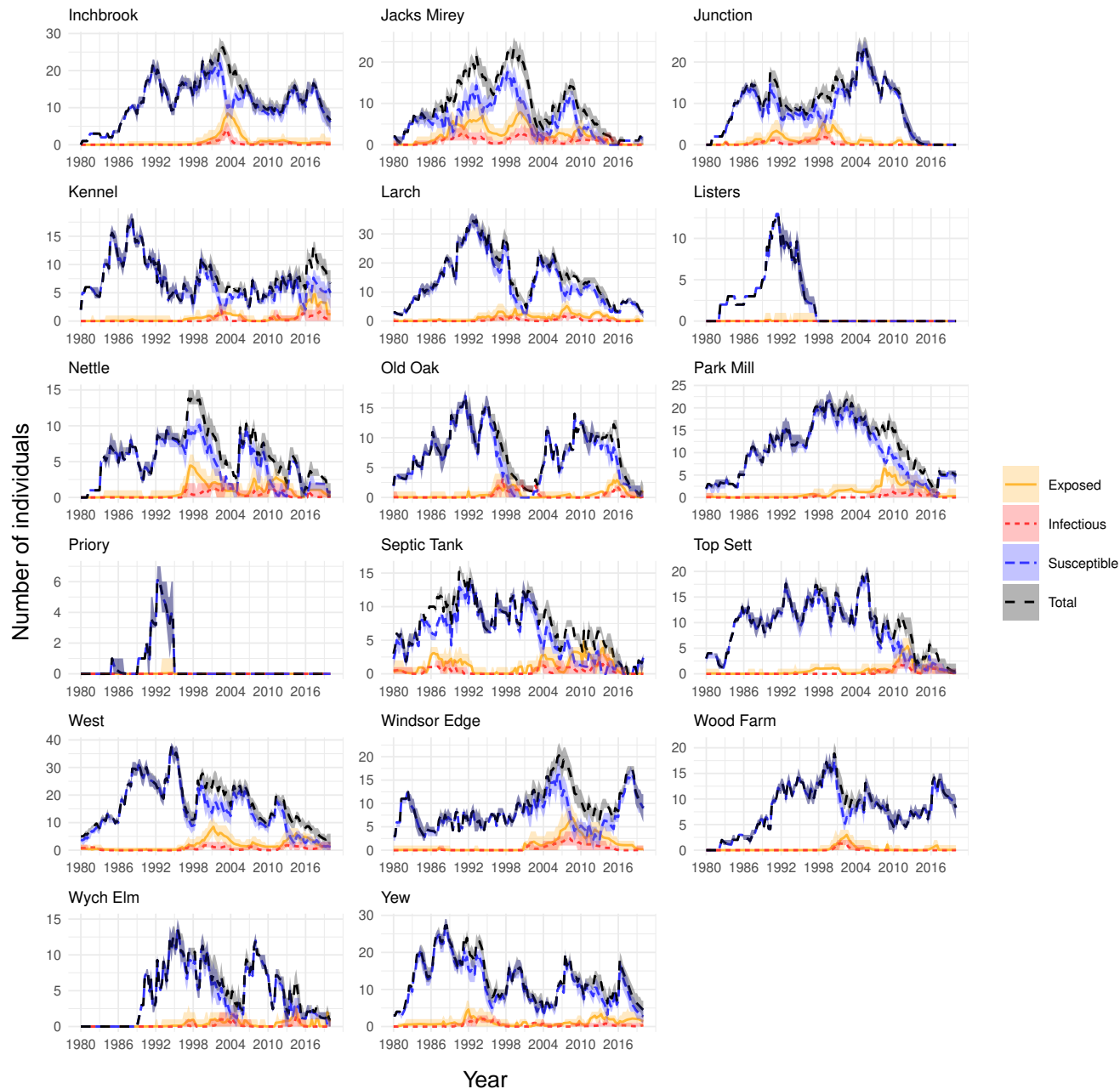

Supplement: S4 Fig — (PDF) [file pcbi.1012592.s005.pdf]

A

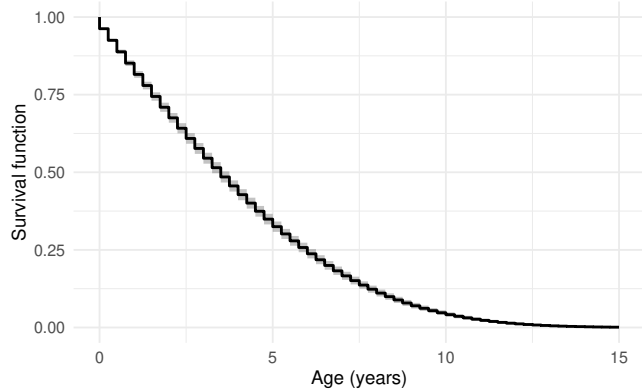

B

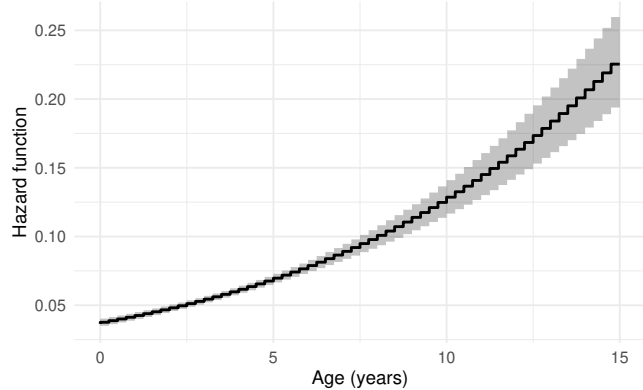

Supplement: S5 Fig — (A) the survival function, and (B) the hazard function. Posterior means are indicated by black lines and 95% credible intervals by grey ribbons. (PDF) [file pcbi.1012592.s006.pdf]
